# The use of genetically humanized animal models for personalized medicine approaches

**DOI:** 10.1242/dmm.041673

**Published:** 2019-10-01

**Authors:** Annemieke Aartsma-Rus, Maaike van Putten

**Affiliations:** Department of Human Genetics, Leiden University Medical Center, Albinusdreef 2, 2333 ZA Leiden, the Netherlands

**Keywords:** Genetic therapies, Genome editing, Exon skipping, Pre-clinical studies, Muscular dystrophy

## Abstract

For many genetic diseases, researchers are developing personalized medicine approaches. These sometimes employ custom genetic interventions such as antisense-mediated exon skipping or genome editing, aiming to restore protein function in a mutation-specific manner. Animal models can facilitate the development of personalized medicine approaches; however, given that they target human mutations and therefore human genetic sequences, scientists rely on the availability of humanized animal models. Here, we outline the usefulness, caveats and potential of such models, using the example of the hDMDdel52/*mdx* model, a humanized model recently generated for Duchenne muscular dystrophy (DMD).

## Abstract (precis)

**Summary:** Personalized medicine approaches benefit from humanized animal models. Here, we outline the usefulness, caveats and considerations for generating and using these models for pre-clinical studies of Duchenne muscular dystrophy.

## Introduction

Genetic mutations underlie thousands of different inherited diseases. With the advance of DNA sequencing approaches, such as massively parallel sequencing (see Box 1, Glossary), it is now feasible to provide a genetic diagnosis for more and more diseases (Lochmüller et al., 2018; Boycott et al., 2019). Gene addition therapy has historically been an obvious choice for genetic diseases caused by the inactivation of a particular gene. Here, the idea is to provide the cells and tissues affected by the mutation with a functioning copy of the missing gene or cDNA to allow production of the missing protein. Recently, progress has also been made for personalized medicine approaches aimed at modifying the disease-causing gene through genome editing (Box 1), or its transcript through modulation of splicing (Box 1) to allow production of the missing protein (Verhaart and Aartsma-Rus, 2019). Most such genetic diseases are caused by a variety of mutations and mutation types, distributed over the gene. As such, these approaches have to be ‘custom made’ to target specific mutations.

**Box 1. Glossary**

**Antisense oligonucleotide (AON):** Small synthetic modified DNA or RNA molecule that is used as a therapeutic modality. AONs act by hybridizing to target transcripts in a sequence-specific manner and can have different effects, e.g. transcript knockdown or splicing modulation.

**Dominant inheritance:** Diseases caused by mutations on one allele of a gene. Often, these involve toxic gain-of-function mutations. When offspring inherits a single copy of the mutation, they will be affected by the disease.

**Genome editing:** Targeted alteration of genomic DNA at a specific location.

**In-frame:** See also reading frame. A mutation is said to be in-frame when it does not disrupt the reading frame. This occurs when the number of nucleotides inserted or deleted is divisible by three. As three nucleotides code for an amino acid, this means an integer number of amino acids is added or lost.

**Massively parallel sequencing:** A high-throughput method for sequencing that allows sequencing of millions of short pieces of DNA simultaneously; also referred to as ‘next generation sequencing’.

**Reading frame:** In the transcript, each set of three nucleotides (codon) encodes a single amino acid. A deletion or insertion of one or more nucleotides (but a number not divisible by three) will cause a shift in the frame. After this mutation, the mRNA will code for the wrong amino acids and the resulting protein will not be functional. Generally, the wrong frame will contain many stop codons, to avoid the cell wasting amino acids on synthesizing a non-functional protein.

**Recessive inheritance:** Diseases in which both alleles of a gene have to be mutated to cause disease.

**Splicing:** The process through which introns are removed from pre-mRNA transcripts and the exons are joined by the splicing machinery to generate an mRNA transcript, which is then translated into a protein.

**Transcriptor activated ligand effector nuclease (TALEN):** A system similar to CRISPR/Cas9 that can be used to generate targeted double-stranded breaks in DNA. TALEN is a two-component system comprising a DNA-binding protein domain, which needs to be customized to the target genetic sequence, and the FokI DNA-cleaving domain. Nowadays, TALEN has been largely replaced by CRISPR/Cas9, as this is easier to work with.

Personalized medicine approaches have to be developed and pre-clinically tested in relevant cell and animal models, which ideally contain the human target sequences. This is important because mutation-specific therapies will not work in mouse models because mice often lack the target human disease-causing sequence owing to species-specific genetic variation.

Generally, patient-derived cell models are obtainable, either as primary cells or immortalized pluripotent stem cells that can be differentiated into the cell type of choice. This allows *in vitro* validation of the personalized medicine approach (Falzarano et al., 2016). However, *in vivo* validation is often more challenging, as it requires humanized models. Those are animal models that carry functioning human genes, cells, tissues and/or organs. Nevertheless, for pre-clinical optimization of personalized medicine approaches, these models are crucial for generation of data that will guide clinical trial design in patients with regards optimal dose, level of functional protein expression required, dosing regimen, route of administration etc. In this Special article, we will illustrate the usefulness, potential, challenges and caveats of humanized animal models for the development of personalized medicine approaches, using the example of the genetic disease Duchenne muscular dystrophy (DMD).

## Personalized medicine for Duchenne muscular dystrophy

DMD is a severe progressive muscle-wasting disease that is characterized by the continuous loss of function, with major disease milestones such as loss of ambulation and the requirement for assisted ventilation occurring at around 12 and 20 years of age, respectively (Birnkrant et al., 2018b). The median survival of DMD patients in the Western world is 30 years, with most patients succumbing to respiratory or cardiac complications (Birnkrant et al., 2018a,b).

**Fig. 1.**
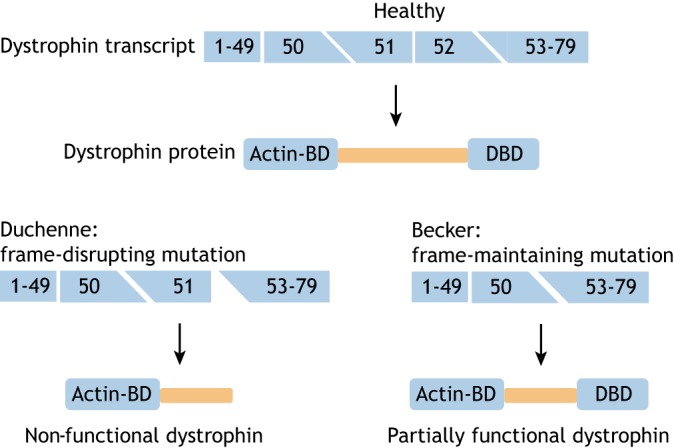
**Dystrophin mutations underlie both Duchenne and Becker muscular dystrophy.** The *DMD* gene encodes the dystrophin protein, which links the actin-cytoskeleton to the extracellular matrix with its actin binding domain (Actin-BD) and dystroglycan binding domain (DBD), respectively (upper panel). In Duchenne patients, mutations, generally deletions involving one or more exons, disrupt the reading frame. In the example in the bottom left panel, an exon 52 deletion causes a frameshift and premature truncation of protein translation, and a non-functional dystrophin. In Becker patients, deletions maintain the reading frame. In the example in the bottom right panel a deletion of exon 51-52 does not disrupt the reading frame, thus allowing the production of an internally deleted dystrophin that contains both crucial domains and that, consequently, is partially functional.

The disease primarily affects men because DMD is caused by mutations in the dystrophin-encoding *DMD* gene that is located on the X chromosome. Dystrophin normally provides stability to contracting muscle by mechanically linking the actin cytoskeleton of muscle fibers to the extracellular matrix (Koenig et al., 1988). Lacking dystrophin, muscle fibers continuously accumulate damage, which leads to chronic inflammation, failed regeneration and gradual replacement of muscle tissue by fibrotic and adipose tissues. Most DMD patients carry a deletion of one or more exons (∼65%) or a duplication of one or more exons (∼10%). These mutations cluster in hotspot regions between exons 2 and 20 and exons 45 and 53. Small mutations (insertions, deletions, nonsense mutations and splice site mutations) account for ∼25% of mutations and occur throughout the gene (Aartsma-Rus et al., 2006). Mutations in the same gene also cause Becker muscular dystrophy (BMD), a disease that is milder compared to DMD. BMD symptoms become apparent later and the disease progression is slower. This discrepancy can be explained by the so-called reading frame (Box 1) rule, posited first by Monaco et al. (1988) (Fig. 1). In DMD patients, reading frame-disrupting mutations or nonsense mutations cause premature truncation of protein translation and unstable, non-functional dystrophin. In BMD patients however, mutations maintain the reading frame, allowing the production of internally deleted but partially functional dystrophin proteins.

**Fig. 2.**
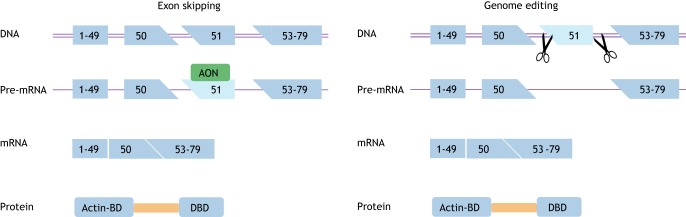
**Schematic depiction of therapeutic exon skipping and genome editing approaches for Duchenne muscular dystrophy.** Exon skipping (left panel) interferes in the pre-mRNA splicing process using antisense oligonucleotides (AON) that target a specific exon (exon 51 in this example). Thus the target exon is hidden from the splicing machinery and ‘skipped’ from the mature mRNA. This enlarges the deletion, but restores the reading frame, thus allowing the production of an internally deleted Becker-like dystrophin. Genome editing (right panel) acts on the DNA level, using guide RNAs that guide the Cas9 enzymes (scissors) to specific locations in the gene. This will result in double-stranded DNA breaks, which are repaired by non-homologous end joining in postmitotic cells (such as skeletal muscle fibers), leading to a larger deletion. Consequently, all transcripts produced have an in-frame deletion, thus allowing the production of internally deleted Becker-like dystrophin. Actin-BD, actin binding domain; DBD, dystroglycan binding domain.

Personalized medicine approaches for DMD stem from the fact that most DMD patients in theory have the genetic capacity to produce BMD-like dystrophins. Restoring the reading frame can occur either at the transcript level through exon skipping or at the DNA level through genome editing (Fig. 2) (Verhaart and Aartsma-Rus, 2019). For exon skipping, small fragments of modified DNA or RNA termed antisense oligonucleotides (AONs, Box 1) specifically recognize a target exon. AON binding renders this target exon inaccessible to the splicing machinery and it is thus not included in the mRNA. This way, the genetic deletion becomes enlarged at the mRNA level, however, because exon skipping restores the reading frame, this mRNA can be translated into shorter, BMD-like dystrophins. Eteplirsen, an AON that induces *DMD* exon 51 skipping, received accelerated approval from the Food and Drug Administration (FDA) in the USA based on minimal increases in dystrophin levels in treated DMD patients (Aartsma-Rus and Krieg, 2017). The FDA has requested the clinical trial sponsor to provide confirmation of functional effects of eteplirsen treatment by 2021, or the approval will be revoked. Clinical trials of exon 45 and exon 53 skipping AONs from various companies are currently ongoing, as is pre-clinical work to optimize AONs targeting additional *DMD* exons and to develop new AON chemistries with improved efficacy (Verhaart and Aartsma-Rus, 2019).

The advantage of this approach is that clinical-grade AONs can be synthetically produced in large amounts and have a relatively small molecular mass, which facilitates systemic delivery. A disadvantage is that owing to the AON, transcript and protein turnover in target cells, AON injections will have to be repeated – patients on eteplirsen currently receive weekly injections.

The clustered regularly interspaced short palindromic repeats (CRISPR)/Cas9 system can be used to modify DNA. Here, guide RNAs lead the Cas9 enzyme to a target region in the DNA where it specifically generates a double-stranded break. This activates the intrinsic cellular DNA repair pathway to correct the break. As muscle fibers are postmitotic, only the error-prone non-homologous end-joining pathway can be activated. However, in this way, the system can be utilized to either remove a target exon from the DNA completely, or to mutate an aberrant splice site (Long et al., 2016; Nelson et al., 2016; Tabebordbar et al., 2016; Hakim et al., 2018). In both cases, the targeted exon will not end up in the mRNA and the reading frame will be restored. The advantage of genome editing is that the treatment needs to occur only once, as each mRNA transcribed from the edited DNA will be in-frame. However, this genome editing approach has currently only been evaluated pre-clinically. Before it can advance to clinical trials for DMD patients, multiple hurdles have to be overcome, involving not only efficient delivery of the guide RNAs and the Cas9 enzyme, but also the potential for off-target effects both in the target cells and, when systemically delivered, also in non-target cells.

Both AON and CRISPR/Cas9 approaches are sequence-specific. Owing to sequence variation between man and mouse, the human *DMD* exon 51 AONs and human guide RNAs will not work in the most commonly used animal model for DMD, the *mdx* mouse. This strain carries a spontaneous truncating mutation in exon 23 of the murine *Dmd* gene (Sicinski et al., 1989). As exon 23 is in-frame (Box 1), skipping or deleting this exon bypasses the aberrant stop codon while maintaining the reading frame. Indeed, treatment with exon 23-skipping AONs or adeno-associated viral vectors containing exon 23-targeting CRISPR/Cas9 restored dystrophin expression in the *mdx* mouse model (Long et al., 2016; Lu et al., 2003; Mann et al., 2002; Nelson et al., 2016; Tabebordbar et al., 2016; Wilton et al., 1999), providing proof-of-principle results for both exon skipping and genome editing therapies. However, these murine exon 23-targeting approaches will not benefit any patients.

This poses a challenge for translation and clinical trial design. When determining active compound doses to test in clinical trials, these will be based on the doses of the exon 23 compounds tested in mice, as researchers know e.g. which AON or CRISPR/Cas9 components concentrations in the target tissues restore dystrophin and which concentrations convey functional effects in the model. However, at least for the exon skipping approach, it is known that some exons are easier to skip than others – although the reasons for this are not yet understood (Aartsma-Rus et al., 2009; Wilton et al., 2007; Heemskerk et al., 2009), and it is not a given that the pre-clinical findings for mouse exon 23 skipping (or deleting) are equally applicable to human exon 51 skipping (or deleting). As such, a model system allowing the testing of human-specific AONs or CRISPR/Cas9 systems, identical to those that would be used in patients in clinical trials, would be of great value. Notably, whereas humanized models allow testing of the on-target efficiency of human-specific CRISPR/Cas9 systems, they do not allow accurate assessment of off-target effects, as only the transgene has been humanized.

## Humanized models for dystrophin reading frame restoration

Humanized animal models for the evaluation of splice modulating or genome editing therapies need to fulfill several criteria. First, the model needs to contain the human target sequence(s). For splicing modulation, cDNA models containing human copies of the (mutated) genes cannot be used, as these transgenes lack introns and therefore splicing does not occur. Similarly, genome editing for DMD involves excising a whole exon, and therefore generally uses guide RNAs that target intronic regions, and likewise cDNA models are not suitable. Secondly, the model needs to recapitulate the protein deficit seen in the patients. For dominantly inherited disorders (Box 1), this can often be achieved by adding a copy of the mutated human gene, or by replacing part of the murine gene with the part of the human gene that contains the mutation. However, for recessive diseases (Box 1), one has to either introduce the human mutation in a homozygous fashion, or add a copy of the mutated human gene and delete or inactivate the second murine allele.

**Fig. 3.**
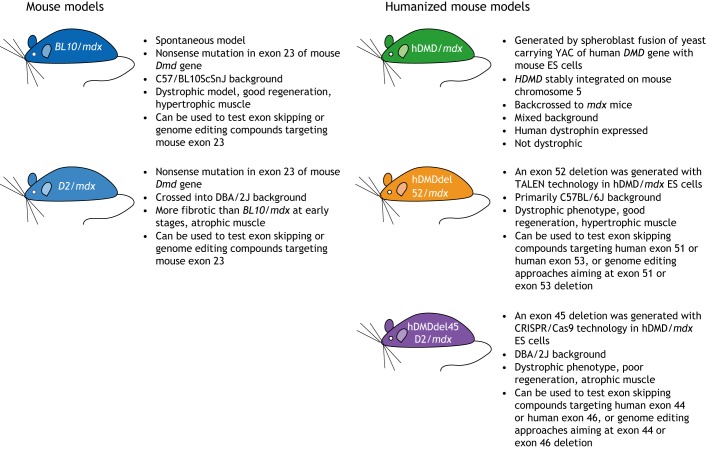
**Overview of the mouse models most commonly used to evaluate genetic therapies.**

Different mouse models with mutations in murine or human dystrophin genes are available for DMD (Fig. 3) (McGreevy et al., 2015). The first humanized model to become available was the hDMD/*mdx* mouse (’t Hoen et al., 2008). This mouse carries copies of the human *DMD* gene integrated into mouse chromosome 5. However, the human *DMD* gene produces human dystrophin, which can functionally compensate for the lack of mouse dystrophin caused by the hallmark *mdx* mutation. As such, the hDMD/*mdx* mouse did not develop a pathology (’t Hoen et al., 2008). Therefore, although this model could be used to validate whether human AONs induce exon skipping (Bremmer-Bout et al., 2004; Arechavala-Gomeza et al., 2007; Popplewell et al., 2010; Heemskerk et al., 2009), it could not be used to assess the effect of AONs on protein production or muscle function.

Thus, our own group and others embarked on generating deletions in the *hDMD* transgene in hDMD/*mdx* embryonic stem cells (Veltrop et al., 2013). We generated the hDMDdel52/*mdx* mouse by using the transcription activator ligand effector nuclease (TALEN; Box 1) system to induce a targeted double-stranded break around exon 52, and homologous recombination with a vector containing a blasticidine resistance gene flanked by homology arms for introns 51 and 52 to generate an exon 52 deletion in the *hDMD* gene of the hDMD/*mdx* mouse (Veltrop et al., 2018). Similarly, Young et al. used Cas9 and guide RNAs targeting introns 44 and 45 to delete exon 45 in the *hDMD* gene, thus generating the hDMDdel45/*mdx* model (Young et al., 2017). The latter was backcrossed to the DBA/2J background, which is known to develop more severe fibrosis at early stages of the DMD disease (Gordish-Dressman et al., 2018; Heydemann et al., 2005; Coley et al., 2016; van Putten et al., 2019).

Both humanized models have a clear dystrophic phenotype with elevated plasma creatine kinase levels, a marker for muscle damage, and centrally nucleated muscle fibers, a marker for active regeneration. Functional analysis of the hDMDdel52/*mdx* mice further revealed deficits in e.g. hanging tests (Veltrop et al., 2018). As expected, the hDMDdel45/*mdx* model, having the DBA/2J background, showed significant levels of fibrosis in skeletal muscles (Young et al., 2017).

These models can be utilized to evaluate therapeutic exon skipping or genome editing approaches using the same AONs or genome editing tools as would be used in humans. For the hDMDdel52/*mdx* model, intramuscular injection of exon 51 and exon 53 AONs resulted in the skipping of the target human exon and dystrophin restoration (Veltrop et al., 2018). For the hDMDdel45/*mdx* model, guide RNAs targeting introns 44 and 55 were used to generate a large secondary in-frame deletion spanning exons 45-55, which restored dystrophin production (Young et al., 2017).

## Further considerations

Notably, the hDMDdel52/*mdx* model was developed while clinical trials of exon 51 and exon 53 skipping AONs were ongoing. This made it impossible to obtain funding to generate the model, as this humanized mouse strain was initially referred to by reviewers and funding agencies as ‘obsolete’. Now that the model is available, however, our personal experience shows that it is in very high demand by both academic collaborators and pharmaceutical companies working on exon skipping or genome editing. When we generated the model, the CRISPR/Cas9 system was not yet available, so we could not have foreseen this as a potential application. Still, the model has added value for further research into exon skipping therapeutic approaches, even though AONs were already in clinical trials.

Although pharmacokinetic and pharmacodynamic data obtained in patients is preferred over data from a model system, because of the invasiveness of the procedures it is impossible to perform multiple skeletal muscle biopsies over time (Verhaart et al., 2019) or to obtain biopsies from the diaphragm and heart in humans. The humanized deletion models now allow researchers to assess the biodistribution of AONs to different skeletal muscles and other tissues, which doses are required to restore which level of dystrophin for different skeletal muscle groups and heart, and the timing and longevity of these effects. Ideally, these data would have been obtained before the clinical trials were set up. Still, collecting them now can help fill some of the knowledge gaps that cannot be filled by data obtained in humans. Furthermore, although an exon 51 skipping drug has been approved by the FDA, it is clear that there is room for improvement, with multiple chemically modified exon 51 AONs in (pre-)clinical development (Verhaart and Aartsma-Rus, 2019; Fletcher et al., 2017). Here, the humanized model provides a way to obtain the pre-clinical data needed to guide future clinical trial design.

Although these humanized models can be a great facilitator of personalized medicine development, they also have some caveats. Because personalized medicine approaches are mutation-dependent, one would theoretically have to develop many different models to perform pre-clinical studies for each and every different AON or genome editing system. This is challenging because of the time and effort involved, not only for generating the model, but also to confirm that the natural history of the model is similar to that of available models and/or reciprocates the human pathology. Furthermore, this tailored mutation-specific approach poses ethical constrains, as it would vastly increase the number of animals used in pre-clinical development. Thus, it is important that researchers carefully select the mutation to be used in the humanized model, e.g. prioritize a common mutation rather than a rare one.

Another important consideration when planning on developing a humanized mouse model stems from reports that some mutations that cause cryptic splicing in humans result in no defect or different defects in mice (Garanto et al., 2015, 2013). Therefore, when generating humanized mouse models to study splice modulation, it is important to validate the genotype in cell models to ensure the intended mutation reciprocates the molecular defect in mouse cells before embarking on generating *in vivo* mouse models.

In summary, humanized animal models can provide great tools to facilitate the development and validation of personalized medicine approaches but, as with all model systems, one has to be aware of the caveats and limitations.
